# Intestine-Specific NHE3 Deletion in Adulthood Causes Microbial Dysbiosis

**DOI:** 10.3389/fcimb.2022.896309

**Published:** 2022-05-26

**Authors:** Jianxiang Xue, Jessica A. Dominguez Rieg, Linto Thomas, James R. White, Timo Rieg

**Affiliations:** ^1^ Department of Molecular Pharmacology and Physiology, University of South Florida, Tampa, FL, United States; ^2^ James A. Haley Veterans’ Hospital, Tampa, FL, United States; ^3^ Resphera Biosciences LLC, Baltimore, MD, United States; ^4^ Center for Hypertension and Kidney Research, University of South Florida, Tampa, FL, United States

**Keywords:** NHE3, microbiome, intestine, inflammatory bowel disease, colitis, dysbiosis, ulcerative colitis

## Abstract

In the intestine, the Na^+^/H^+^ exchanger 3 (NHE3) plays a critical role for Na^+^ and fluid absorption. NHE3 deficiency predisposes patients to inflammatory bowel disease (IBD). In mice, selective deletion of intestinal NHE3 causes various local and systemic pathologies due to dramatic changes in the intestinal environment, which can influence microbiota colonization. By using metagenome shotgun sequencing, we determined the effect of inducible intestinal epithelial cell-specific deletion of NHE3 (NHE3^IEC-KO^) in adulthood on the gut microbiome in mice. Compared with control mice, NHE3^IEC-KO^ mice show a significantly different gut microbiome signature, with an unexpected greater diversity. At the phylum level, NHE3^IEC-KO^ mice showed a significant expansion in *Proteobacteria* and a tendency for lower *Firmicutes/Bacteroidetes* (F/B) ratio, an indicator of dysbiosis. At the family level, NHE3^IEC-KO^ mice showed significant expansions in *Bacteroidaceae*, *Rikenellaceae*, *Tannerellaceae*, *Flavobacteriaceae* and *Erysipelotrichaceae*, but had contractions in *Lachnospiraceae*, *Prevotellaceae* and *Eubacteriaceae*. At the species level, after removing those with lowest occurrence and abundance, we identified 23 species that were significantly expanded (several of which are established pro-inflammatory pathobionts); whereas another 23 species were found to be contracted (some of which are potential anti-inflammatory probiotics) in NHE3^IEC-KO^ mice. These results reveal that intestinal NHE3 deletion creates an intestinal environment favoring the competitive advantage of inflammophilic over anti-inflammatory species, which is commonly featured in conventional NHE3 knockout mice and patients with IBD. In conclusion, our study emphasizes the importance of intestinal NHE3 for gut microbiota homeostasis, and provides a deeper understanding regarding interactions between NHE3, dysbiosis, and IBD.

## Introduction

The Na^+^/H^+^ exchanger 3 (NHE3) mediates Na^+^ and fluid absorption in the intestine and reabsorption in the kidney. Knockout of NHE3 selectively in the small intestine and colon of mice results in disruption of intestinal structural integrity, persistent alkaline diarrhea, metabolic acidosis, hyponatremia and hyperkalemia associated with drastically elevated plasma aldosterone levels, and increased mortality rate (Xue et al., 2020; Xue et al., 2022). Together, these symptoms are consistent with patients experiencing congenital sodium diarrhea (CSD).

In patients, germline mutations in guanylyl cyclase C, a regulator of NHE3, and in NHE3 itself result in CSD (Janecke et al., 2015; Janecke et al., 2016). Of note, a number of patients with these mutations develop inflammatory bowel disease (IBD), implying that NHE3 dysfunction directly or indirectly predisposes patients to IBD (Anderson et al., 2011; Jostins et al., 2012). Several studies have demonstrated an inverse relationship between NHE3 activity and pathogenesis of IBD. Conventional NHE3 knockout (NHE3^-/-^) mice develop spontaneous distal colitis (Laubitz et al., 2008) and are highly susceptible to dextran sodium sulfate (DSS)-induced mucosal injury (Kiela et al., 2009). Similarly, intestinal NHE3 dysfunction has been observed in other murine colitis models such as interleukin (IL) 2-deficient mice (Barmeyer et al., 2004), IL10-deficient mice (Lenzen et al., 2012), and mice treated with DSS or trinitrobenzene sulfate acid (Sullivan et al., 2009). Development of IBD is thought to occur as a result of pathologic interactions between the intestinal environment, immune system, and microbial factors in a genetically susceptible host. Several studies have shown that NHE3 deficiency predispose patients and animals to microbial dysbiosis and IBD (Engevik et al., 2013; Larmonier et al., 2013; Janecke et al., 2016; Laubitz et al., 2016; Harrison et al., 2018). NHE3^-/-^ mice show reduced microbial diversity with expansion of the inflammation-associated *Bacteroidetes* phylum and contraction of the *Firmicutes* phylum (Engevik et al., 2013; Harrison et al., 2018), which is consistent with what has been found in IBD patients (Ott et al., 2004; Sokol and Seksik, 2010; Fava and Danese, 2011). When NHE3^-/-^ mice were re-derived into a germ-free facility, they exhibited no inflammatory phenotype and show a delayed mortality in response to DSS (Larmonier et al., 2013). On the other hand, reintroduction of conventional microflora in germ-free NHE3^-/-^ mice restored spontaneous distal colitis, highlighting the role of microbiota in NHE3 dysfunction-induced colitis (Larmonier et al., 2013). It should be noted that colonization of microbiota begins at birth; thus, studies utilizing NHE3^-/-^ mice are evaluating the effect of NHE3 deletion on the gut microbiome across the entire development span. However, it is not known if lack of NHE3 in adulthood exerts similar effects on gut microbiome.

The aim of the current study was to determine how inducible intestinal-specific knockout of NHE3 in adulthood impacts the gut microbiome. Our data demonstrate that functional NHE3 is required for a healthy microbiome, and lack of intestinal NHE3 results in microbial changes that favor inflammation and downstream pathological consequences.

## Materials and Methods

### Animals

All animal experimentation was conducted in accordance with the Guide for Care and Use of Laboratory Animals (National Institute of Health, Bethesda, MD) and was approved by the Institutional Animal Care and Use Committee (3338R). The generation of tamoxifen-inducible intestinal specific NHE3 knockout mice (NHE3^IEC-KO^) has been described previously (Xue et al., 2020). Mice were genotyped by polymerase chain reaction from genomic DNA isolated from ear punch. At 11 weeks of age, NHE3 deletion was induced in mice by application of tamoxifen (67 mg/kg), initially dissolved in 5% (v/v) of ethanol followed by adding 95% (v/v) of corn oil. Tamoxifen was administered *via* oral gavage (volume 1% of body weight) for 5 consecutive days to control and NHE3^IEC-KO^ mice. Only male mice were used for microbiome analysis.

### Sample Collection, and Shotgun Metagenomic Sequencing and Quality Control of Reads

At 12 weeks of age, mice (*n* = 9/genotype) were single housed for 2 weeks and colon content was collected into collection tubes containing DNA stabilization buffer. Whole metagenome shallow shotgun sequencing (at least 2 million paired-end reads/sample) was performed using the Illumina miSeq or Illumina NextSeq instrument (the instrument used is dependent on the number of samples in a batch, Illumina, San Diego, CA). For the analysis, samples were extracted using the Qiagen PowerMag Microbiome DNA Isolation kit (Hilden, Germany) on the King Fisher automated platform (Thermo Scientific, Waltham, MA). Isolated DNA was quantitated using a fluorescent concentration assay and normalized to prepare for library prep using the Illumina Nextera XT DNA Library prep recommendations. Runs were spiked with 1% PhiX. Standard processing used 2 x 150 base pair paired-end sequencing with dual 8 base pair indexes. The instrument run takes ~29 hours. Criteria for acceptable results: final run must have a cluster density of 180-230K/mm^2^ with greater than 80% of clusters passing filter, and at least 75% of bases must call at a minimum Phred score of Q30 (99.5%).

### Data Analysis and Statistics

The One Codex Database consists of ~114,000 complete microbial genomes, including 62,000 distinct bacterial genomes, 48,000 viral genomes, and approximately 4,000 fungal, archaeal, and eukaryotic genomes. The human genome was included to screen out host reads, and a complete list of references is available in the One Codex application at https://app.onecodex.com/references. The database is assembled from both public and private sources, with a combination of automated and manual curation steps to remove low quality or mislabeled records. Comparing a microbial sample against the One Codex Database consists of three sequential steps. First, every individual NGS read was compared against the One Codex Database by exact alignment using k-mers where k=31 [(Ames et al., 2013; Wood and Salzberg, 2014) for details on k-mer based classification]. The k-mer classification results were filtered based on the relative frequency of unique k-mers in the sample, sequencing artifacts were filtered out of the sample. This filtering only removes probable sequencing or reference genome-based artifacts and does not filter out low abundance or low confidence hits. Finally, the relative abundance of each microbial species was estimated based on the depth and coverage of sequencing across every available reference genome. Microbial profiles were generated using the OneCodex analysis platform using the targeted loci module with summarization at the phylum through species levels. Results were normalized to an even level of coverage through subsampling without replacement (11,000 observations per sample). Statistical analyses were performed using the R Statistical Environment (v.3.5.3). Normalized species level profiles were utilized to calculate α- and β-diversity measures with the vegan R package. Principal coordinates analysis (PCoA) was performed using the ape R package. PERMANOVA calculations were executed using the *adonis* function in vegan. Differential abundance analysis included calculation of the Mann-Whitney U test and Welch’s t-test with log10 transformed values. Data from **Table 1** with false discovery rate p-value adjustment are shown as **Supplementary Table 1**. Unsupervised clustering with heatmap overlay applied the heatmap function in R with the Euclidean distance metric. Linear discriminant analysis (LDA) effect size (LEfSe) (Segata et al., 2011) was applied to normalized taxonomic data to compare taxonomic membership between genotypes, followed by visualization of identified taxa by cladogram and individual LDA scores. Additional visualizations of stacked histograms and boxplots utilized the ggplot2 package in R. Aggregation of abundance estimates for aerobic, facultative anaerobe, and obligate anaerobe categories applied a taxonomic classification strategy based on literature review in which (1) aerobic included members of *Pseudomonas*, *Mycobacterium*, *Nocardia*, *Bacillus* or *Streptomyces*, (2) those not assigned to aerobic that were members of the phyla *Actinobacteria*, *Firmicutes*, *Proteobacteria* were assigned as facultative anaerobes, and otherwise (3) obligate anaerobes were members matching the taxa *Tenericutes*, *Bacteroidetes*, *Actinomyces*, *Bacteroides*, *Clostridium*, *Fusobacterium*, *Peptostreptococcus*, *Porphyromonas*, *Prevotella*, *Propionibacterium*, and *Veillonella*.

**Table 1:** Differential abundance of microbiota across taxonomic levels between NHE3^IEC-KO^ and control mice.

Taxa	Taxonomic description	Relative abundance (% ± SEM)		P value	Change (KO vs Con)
		KO (n=9)	Con (n=9)		
Phylum	*Proteobacteria*	11.34 ± 1.65	5.85 ± 0.67	0.00454	↑
	*Bacteroidetes*	65.08 ± 2.11	58.62 ± 5.78	0.24768	—
	*Firmicutes*	20.94 ± 1.40	33.15 ± 5.57	0.10450	—
	*Firmicutes/Bacteroidetes*	0.33 ± 0.03	0.74 ± 0.20	0.07860	—
Class	*Alphaproteobacteria*	1.79 ± 0.40	0.61 ± 0.06	0.00030	↑
	*Deltaproteobacteria*	2.07 ± 0.32	1.14 ± 0.21	0.02620	↑
	*Erysipelotrichia*	0.78 ± 0.33	0.10 ± 0.02	0.01404	↑
	*Flavobacteriia*	1.64 ± 0.05	1.08 ± 0.12	0.00829	↑
	*Clostridia*	14.94 ± 1.37	28.01 ± 5.34	0.05870	—
	*Bacteroidia*	60.14 ± 2.12	54.67 ± 5.39	0.28030	—
	*Epsilonproteobacteria*	4.15 ± 1.30	1.23 ± 0.29	0.07641	—
Order	*Erysipelotrichales*	0.79 ± 0.32	0.09 ± 0.02	0.00914	↑
	*Cytophagales*	0.72 ± 0.09	0.47 ± 0.05	0.02629	↑
	*Flavobacteriales*	1.66 ± 0.04	1.10 ± 0.12	0.00880	↑
	*Rhizobiales*	0.79 ± 0.14	0.41 ± 0.03	0.00711	↑
	*Clostridiales*	14.59 ± 1.38	27.71 ± 5.36	0.05696	—
	*Campylobacterales*	4.09 ± 1.28	1.21 ± 0.29	0.07963	—
	*Bacteroidales*	59.37 ± 2.08	54.15 ± 5.37	0.29107	—
Family	*Bacteroidaceae*	15.73 ± 1.43	10.49 ± 1.72	0.01911	↑
	*Erysipelotrichaceae*	0.79 ± 0.32	0.09 ± 0.02	0.00575	↑
	*Flavobacteriaceae*	1.44 ± 0.04	0.90 ± 0.10	0.00533	↑
	*Rikenellaceae*	4.05 ± 0.64	1.49 ± 0.24	0.00020	↑
	*Tannerellaceae*	6.03 ± 0.35	4.29 ± 0.48	0.02701	↑
	*Lachnospiraceae*	7.52 ± 0.96	18.59 ± 4.16	0.02111	↓
	*Eubacteriaceae*	0.87 ± 0.12	2.00 ± 0.41	0.02191	↓
	*Prevotellaceae*	5.91 ± 0.37	13.45 ± 2.33	0.00383	↓
	*Ruminococcaceae*	1.53 ± 0.47	1.23 ± 0.21	0.99495	—
	*Barnesiellaceae*	9.39 ± 0.98	8.33 ± 1.07	0.41859	—
	*Muribaculaceae*	7.13 ± 0.75	5.87 ± 0.83	0.25659	—
Genus	*Bacteroides*	15.70 ± 1.44	10.46 ± 1.72	0.01897	↑
	*Enterococcus*	1.31 ± 0.09	1.02 ± 0.07	0.03532	↑
	*Alistipes*	3.85 ± 0.65	1.46 ± 0.24	0.00037	↑
	*Parabacteroides*	4.98 ± 0.28	3.59 ± 0.37	0.02994	↑
	*Robinsoniella*	0.03 ± 0.01	0.42 ± 0.12	0.00004	↓
	*Eubacterium*	0.87 ± 0.12	1.99 ± 0.41	0.02509	↓
	*Kineothrix*	0.28 ± 0.05	0.99 ± 0.28	0.01751	↓
	*Roseburia*	1.59 ± 0.22	5.72 ± 1.37	0.00527	↓
	*Prevotella*	5.05 ± 0.34	12.46 ± 2.14	0.00199	↓
	*Clostridium*	1.86 ± 0.05	1.55 ± 0.12	0.05337	—
	*Butyrivibrio*	0.58 ± 0.07	1.09 ± 0.22	0.09220	—
	*Lachnoclostridium*	2.85 ± 0.39	5.54 ± 1.16	0.06762	—
	*Muribaculum*	7.12 ± 0.75	5.89 ± 0.81	0.27338	—
Species	*B. thetaiotaomicron*	2.48 ± 0.43	1.04 ± 0.21	0.00287	↑
	*B. nealsonii*	0.23 ± 0.02	0.17 ± 0.02	0.04783	↑
	*B. subtilis*	0.69 ± 0.04	0.53 ± 0.04	0.01438	↑
	*B. fragilis*	1.99 ± 0.12	1.49 ± 0.18	0.04010	↑
	*B. helcogenes*	2.15 ± 0.28	1.23 ± 0.21	0.01559	↑
	*C. baratii*	1.41 ± 0.06	1.13 ± 0.10	0.03734	↑
	*M. massiliensis*	0.23 ± 0.04	0.10 ± 0.03	0.02771	↑
	*F. columnare*	0.11 ± 0.01	0.06 ± 0.01	0.04997	↑
	*R. anatipestifer*	0.19 ± 0.02	0.10 ± 0.02	0.00521	↑
	*R. biformata*	0.24 ± 0.04	0.15 ± 0.02	0.02190	↑
	*H. cetorum*	0.20 ± 0.07	0.00 ± 0.00	0.00262	↑
	*H. ganmani*	0.92 ± 0.30	0.00 ± 0.00	0.00127	↑
	*L. mesenteroides*	0.25 ± 0.02	0.15 ± 0.01	0.00164	↑
	*D. orientale*	0.24 ± 0.02	0.14 ± 0.02	0.00580	↑
	*P. putida*	0.12 ± 0.02	0.04 ± 0.01	0.00352	↑
	*A. finegoldii*	1.48 ± 0.26	0.46 ± 0.09	0.00015	↑
	*A. obesi*	0.21 ± 0.07	0.03 ± 0.01	0.00026	↑
	*A. putredinis*	0.16 ± 0.03	0.05 ± 0.01	0.00165	↑
	*A. senegalensis*	0.17 ± 0.04	0.07 ± 0.02	0.01109	↑
	*A. shahii*	1.05 ± 0.15	0.51 ± 0.07	0.00312	↑
	*S. fumaroxidans*	0.10 ± 0.01	0.04 ± 0.01	0.00820	↑
	*P. distasonis*	4.69 ± 0.29	3.31 ± 0.34	0.02383	↑
	*T. forsythia*	0.85 ± 0.08	0.61 ± 0.09	0.04271	↑
	*B. stercoris*	0.01 ± 0.01	0.23 ± 0.05	0.00011	↓
	*B. thuringiensis*	0.12 ± 0.02	0.24 ± 0.05	0.01937	↓
	*E. eligens*	0.74 ± 0.13	1.71 ± 0.39	0.03761	↓
	*E. plexicaudatum*	0.03 ± 0.01	0.10 ± 0.03	0.04299	↓
	*H. apodemus*	0.00 ± 0.00	0.11 ± 0.05	0.00266	↓
	*H. typhlonius*	0.03 ± 0.02	0.11 ± 0.03	0.00428	↓
	*E. massiliensis*	0.08 ± 0.01	0.29 ± 0.07	0.00099	↓
	*K. alysoides*	0.28 ± 0.05	0.98 ± 0.29	0.01851	↓
	*C. polysaccharolyticum*	0.02 ± 0.01	0.11 ± 0.04	0.02635	↓
	*C. scindens*	0.39 ± 0.08	1.33 ± 0.32	0.00195	↓
	*L. phytofermentans*	0.26 ± 0.03	1.02 ± 0.24	0.00098	↓
	*R. peoriensis*	0.02 ± 0.01	0.41 ± 0.13	0.00003	↓
	*R. hominis*	1.54 ± 0.21	5.62 ± 1.34	0.00458	↓
	*L. ruminis*	0.03 ± 0.02	0.11 ± 0.04	0.02541	↓
	*L. salivarius*	0.02 ± 0.01	0.13 ± 0.05	0.00483	↓
	*P. xylaniphila*	0.05 ± 0.01	0.21 ± 0.05	0.03351	↓
	*P. buccalis*	0.01 ± 0.01	0.20 ± 0.05	0.00141	↓
	*P. conceptionensis*	0.11 ± 0.03	0.25 ± 0.03	0.02915	↓
	*P. dentalis*	0.75 ± 0.06	2.73 ± 0.58	0.00091	↓
	*P. denticola*	0.62 ± 0.07	1.79 ± 0.31	0.00020	↓
	*P. intermedia*	0.40 ± 0.05	1.02 ± 0.19	0.00300	↓
	*P. loescheii*	0.14 ± 0.02	0.44 ± 0.09	0.00348	↓
	*P. ruminicola*	0.43 ± 0.06	0.89 ± 0.14	0.01271	↓
	*B. viscericola*	8.73 ± 0.93	7.54 ± 0.93	0.36928	—
	*M. intestinale*	7.14 ± 0.74	5.85 ± 0.83	0.24582	—

the symbol “↑” indicates expansion; “↓” indicates contraction in NHE3^IEC-KO^ compared with control mice; “—” indicates no difference between genotypes. Unadjusted t-test P values are shown.

## Results

### Intestinal NHE3 Deletion in Adulthood Distinctly Impacts Microbiome Diversity

**Figure 1:**
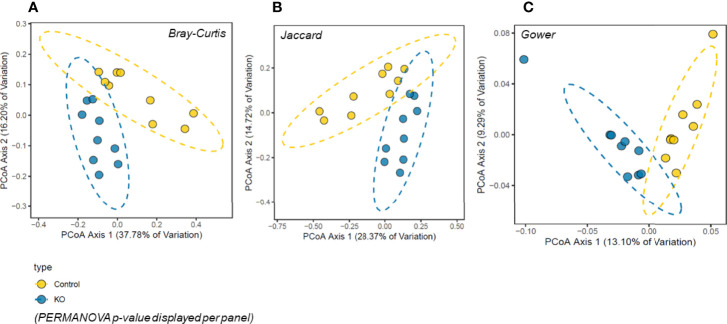
β-diversity principal coordinates analysis (PCoA) in NHE3^IEC-KO^ and control mice. Microbiota composition was significantly different between the two genotypes (*n* = 9/genotype) by three different measures of beta-diversity: **(A)** Bray-Curtis, **(B)** Jaccard, and **(C)** Gower distances (PERMANOVA p-value displayed per panel). Percentage of variation explained per PCoA axis is displayed with the axis title.

**Figure 2:**
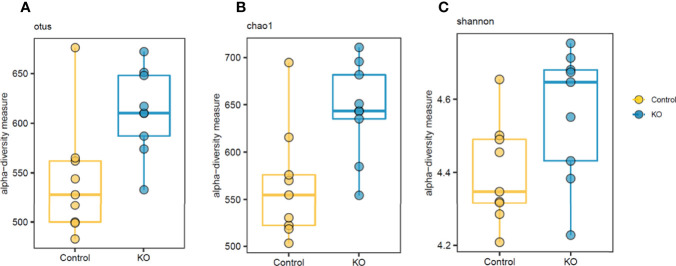
Differential α-diversity levels in NHE3^IEC-KO^ and control mice. α-diversity analysis suggests significant differences in otus **(A)** and chao1 **(B)** richness estimators and Shannon diversity (*P* < 0.05) **(C)** between NHE3^IEC-KO^ and control mice (Mann-Whitney test). *N* = 9/genotype.

The analysis of gut microbiome β-diversity, a measure of diversity differences between genotypes, identified that NHE3^IEC-KO^ mice have a distinct gut microbiome signature that clusters differently when compared with their age- and sex-matched control counterparts. Pairwise compositional dissimilarity analysis using Bray-Curtis (*P* = 0.0002), Jaccard (*P* = 0.0002), and Gower (*P* = 0.0002) indices each showed significant differences between genotypes (**Figure 1**). Further analysis of microbial α-diversity, a measure of variance within a specific genotype, showed that NHE3^IEC-KO^ and control mice harbor distinct populations of gut microbes. Operational taxonomic units (OTU richness, taxa level, *P* = 0.009), Chao1 (richness estimator, phylum level, *P* = 0.007), and Shannon index (reflects species numbers and evenness of species abundance, *P* = 0.046) all showed a significantly greater microbial diversity in NHE3^IEC-KO^ compared with control mice (**Figure 2**).

### Differential Abundance of Microbiota Between NHE3^IEC-KO^ and Control Mice

**Figure 3:**
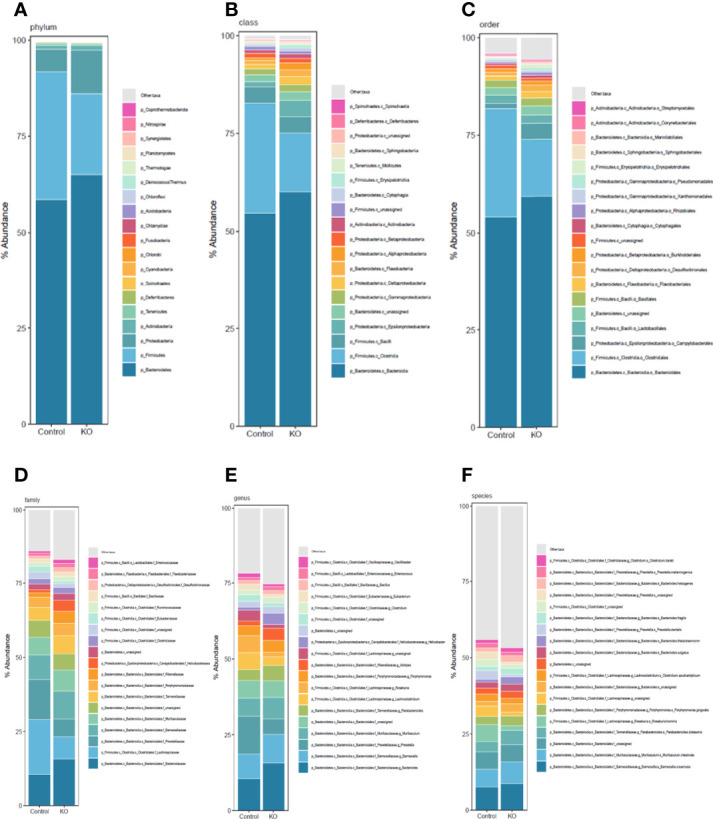
Taxonomic composition distribution histograms of gut microbiota from NHE3^IEC-KO^ and control mice. Composition of gut microbiota at the phylum **(A)**, class **(B)**, order **(C)**, family **(D)**, genus **(E)**, and species **(F)** levels, from NHE3^IEC-KO^ and control mice. *N* = 9/genotype.

We systematically analyzed the microbial composition distribution and differential abundance from phyla down to species levels between NHE3^IEC-KO^ and control mice. Compared with control mice, NHE3^IEC-KO^ mice showed significant differential distribution across taxonomic levels (**Figure 3**). At the phyla level, the most abundant phylum was *Bacteroidetes*, which showed no significant difference between the genotypes (65 ± 2% vs 59 ± 6%, *NS*) (**Table 1**). The second most abundant phylum, *Firmicutes*, showed a tendency to be lower in NHE3^IEC-KO^ mice compared with control mice (21 ± 1% vs 33 ± 6%, *P* = 0.062). The *Firmicutes/Bacteroidetes* (F/B) ratio is widely regarded as an indicator of dysbiosis, with a decreased F/B ratio observed in IBD. We found that the F/B ratio in NHE3^IEC-KO^ mice trended to be lower than in control mice (0.33 ± 0.03 vs 0.74 ± 0.20, *P* = 0.079). Abundance of the phylum *Proteobacteria*, commonly considered to be inflammophilic pathobionts, was nearly 2-fold higher in NHE3^IEC-KO^ mice compared with control mice (11 ± 2% vs 6 ± 1%, *P* < 0.01) (**Table 1**).

At the class level, the greater abundance of *Alphaproteobacteria* (1.8 ± 0.4% vs 0.6 ± 0.1%, *P* < 0.01), *Deltaproteobacteria* (2.0 ± 0.3% vs 1.1 ± 0.2%, *P* < 0.05) and *Epsilonproteobacteria* (4.2 ± 1.3% vs 1.2 ± 0.3%, *P* = 0.076) are primarily responsible for the 2-fold expansion of the phylum *Proteobacteria* in NHE3^IEC-KO^ compared with control mice (**Table 1**). In addition, *Flavobacteriia* (1.6 ± 0.1% vs 1.1 ± 0.1%, *P* < 0.01) and *Erysipelotrichia* (0.8 ± 0.3% vs 0.1 ± 0.1%, *P* < 0.05) also showed significantly greater abundance in NHE3^IEC-KO^ compared with control mice. In contrast, NHE3^IEC-KO^ mice showed a tendency for contraction in *Clostridia* (15 ± 1% vs 28 ± 5%, *P* = 0.059) compared with control mice. However, the most abundant class, *Bacteroidia*, (60 ± 2% vs 55 ± 5%, *NS*) showed no difference between genotypes (**Table 1**).

The most abundant order is *Bacteroidales* (59 ± 2% vs 54 ± 5%, *NS*), which showed no difference between genotypes. NHE3^IEC-KO^ mice showed expansion of *Erysipelotrichales* (0.79 ± 0.3% vs 0.09 ± 0.1%, *P* < 0.01), *Cytophagales* (0.72 ± 0.1% vs 0.47 ± 0.1%, *P* < 0.05), *Flavobacteriales* (1.7 ± 0.1% vs 1.1 ± 0.1%, *P* < 0.01), and *Rhizobiales* (0.79 ± 0.1% vs 0.41 ± 0.1%, *P* < 0.01), and a tendency for increased *Campylobacterales* (4.0 ± 1.3% vs 1.2 ± 0.3%, *P* = 0.079). In contrast, NHE3^IEC-KO^ mice had a tendency for contraction in the order *Clostridiales* (15 ± 1% vs 28 ± 5%, *P* = 0.057) (**Table 1**).

At the family level, significant expansions in the abundance of *Bacteroidaceae* (16 ± 1% vs 11 ± 2%, *P* < 0.05), *Rikenellaceae* (4.0 ± 0.6% vs 1.5 ± 0.2%, *P* < 0.01), *Tannerellaceae* (6.0 ± 0.4% vs 4.3 ± 0.5%, *P* < 0.05), *Flavobacteriaceae* (1.4 ± 0.1% vs 0.9 ± 0.1%, *P* < 0.01) and *Erysipelotrichaceae* (0.8 ± 0.3% vs 0.1 ± 0.1%, *P* < 0.01) were observed in NHE3^IEC-KO^ compared with control mice (**Table 1**); whereas, significant contractions in *Lachnospiraceae* (8 ± 1% vs 19 ± 4%, *P* < 0.05), *Prevotellaceae* (6 ± 1% vs 14 ± 2%, *P* < 0.01) and *Eubacteriaceae* (0.9 ± 0.1% vs 2.0 ± 0.4%, *P* < 0.05) were seen in NHE3^IEC-KO^ compared with control mice. The abundance of *Barnesiellaceae* (9 ± 1% vs 8 ± 1%, *NS*), *Muribaculaceae* (7 ± 1% vs 6 ± 1%, *NS*) and *Ruminococcaceae* (1.5 ± 0.5% vs 1.2 ± 0.2%, *NS*) showed no significant difference between genotypes (**Table 1**).

At the genus level, NHE3^IEC-KO^ mice showed significant expansions in *Bacteroides* (16 ± 1% vs 11 ± 2%, *P* < 0.05), *Parabacteroides* (5.0 ± 0.3% vs 3.6 ± 0.4%, *P* < 0.05), *Alistipes* (3.9 ± 0.7% vs 1.5 ± 0.2%, *P* < 0.01), and *Enterococcus* (1.3 ± 0.1% vs 1.0 ± 0.1%, *P* < 0.05) compared with control mice. In contrast, contractions in *Robinsoniella* (0.03 ± 0.01% vs 0.4 ± 0.1%, *P* < 0.01), *Prevotella* (5 ± 1% vs 13 ± 2%, *P* < 0.01), *Roseburia* (1.6 ± 0.2% vs 5.7 ± 1.4%, *P* < 0.01), *Eubacterium* (0.9 ± 0.1% vs 2.0 ± 0.4%, *P* < 0.05), and *Kineothrix* (0.3 ± 0.1% vs 1.0 ± 0.3%, *P* < 0.05) were observed in NHE3^IEC-KO^ compared with control mice. In addition, NHE3^IEC-KO^ mice showed a tendency for contractions in *Lachnoclostridium* (2.9 ± 0.4% vs 5.5 ± 1.2%, *P* = 0.068) and *Butyrivibrio* (0.6 ± 0.1% vs 1.1 ± 0.2%, *P* = 0.092) and an expansion in *Clostridium* (1.9 ± 0.1% vs 1.6 ± 0.1%, *P* = 0.053). There were no differences in *Muribaculum* between genotypes (7.1 ± 0.8% vs 5.9 ± 0.8%, *NS*) (**Table 1**).

Further microbiome analysis at the species level showed that, after removing the species with lowest occurrence and abundance as well as those unassigned, at least 46 species had significant differential changes in NHE3^IEC-KO^ compared with control mice, with 23 species being expanded and 23 species being contracted in NHE3^IEC-KO^ mice (**Table 1**). A large portion of the species that were expanded in NHE3^IEC-KO^ mice are well-established pathobionts, e.g., *B. thetaiotaomicron* (2.5 ± 0.4% vs 1.0 ± 0.2%, *P* < 0.01), *P. distasonis* (4.7 ± 0.3% vs 3.3 ± 0.3%, *P* < 0.05), *H. ganmani* (0.9 ± 0.3% vs 0 ± 0%, *P* < 0.01), *C. baratii* (1.4 ± 0.1% vs 1.1 ± 0.1%, *P* < 0.05), *H. cetorum* (0.2 ± 0.1% vs 0 ± 0%, *P* < 0.01), *P. putida* (0.1 ± 0.02% vs 0.04 ± 0.01%, *P* < 0.01), *R. anatipestifer* (0.2 ± 0.02% vs 0.1 ± 0.02%, *P* < 0.01), and *F. columnare* (0.1 ± 0.01% vs 0.06 ± 0.01%, *P* < 0.05). In contrast, some potential anti-inflammatory probiotic bacteria are contracted in NHE3^IEC-KO^ mice, including *R. hominis* (1.5 ± 0.2% vs 5.6 ± 1.3%, *P* < 0.01), *E. eligens* (0.7 ± 0.1% vs 1.7 ± 0.4%, *P* < 0.05), *C. scindens* (0.4 ± 0.1% vs 1.3 ± 0.3%, *P* < 0.01), *P. loescheii* (0.1 ± 0.02% vs 0.4 ± 0.09%, *P* < 0.01), *K. alysoides* (0.3 ± 0.1% vs 1.0 ± 0.3%, *P* < 0.05), *L. salivarius* (0.02 ± 0.01% vs 0.13 ± 0.05%, *P* < 0.01), *L. ruminis* (0.03 ± 0.02% vs 0.11 ± 0.04%, *P* < 0.05), and *E. plexicaudatum* (0.03 ± 0.01% vs 0.1 ± 0.03%, *P* < 0.05). Interestingly, two pathogenic species, *P. buccalis* (0.01 ± 0.01% vs 0.2 ± 0.05%, *P* < 0.01) and *H. typhlonius* (0.03 ± 0.02% vs 0.11 ± 0.03%, *P* < 0.01), which were reported to be pro-inflammatory, showed significant contractions in NHE3^IEC-KO^ mice, whereas *B. subtilis* (0.7 ± 0.04% vs 0.5 ± 0.04%, *P* < 0.05), an ideal multifunctional probiotic, was enriched in NHE3^IEC-KO^ mice.

**Figure 4:**
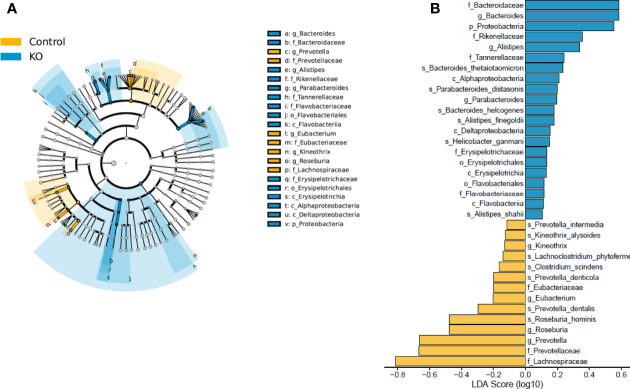
LEfSe analysis of gut microbiota from NHE3^IEC-KO^ and control mice. Cladograms **(A)** shows the microbial clades with the greatest differences in abundance in microbiota from NHE3^IEC-KO^ and control mice. LDA scores **(B)** of microbial clades differing in abundance between NHE3^IEC-KO^ and control mice (with LDA score >0.1 and significance of *P* < 0.05, determined using Kruskal-Wallis test). *N* = 9/genotype.

**Figure 5:**
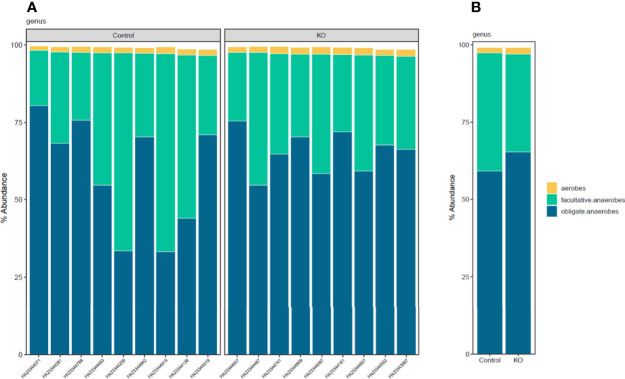
Distribution histograms of aerobic and anaerobic microbial communities from NHE3^IEC-KO^ and control mice. Aerobic/anaerobic microbial communities profiling **(A)** by samples, **(B)** by genotype groups, in NHE3^IEC-KO^ and control mice. *N* = 9/genotype.

Linear discriminant analysis effect size (LEfSe) identified microbiota with the greatest differences in abundance between NHE3^IEC-KO^ and control mice. The family *Bacteroidaceae*, genus *Bacteroides*, and phylum *Proteobacteria* (LDA >0.5) are the representative taxa with significant expansions; whereas the family *Lachnospiraceae*, family *Prevotellaceae* and genus *Prevotella* (LDA >0.5) are the representative taxa with significant contraction in NHE3^IEC-KO^ compared with control mice (**Figure 4**). However, in our LEfSe analysis the LDA scores were low (LDA score <1) because all significant differences were highly correlated. In addition, NHE3^IEC-KO^ mice showed no significant differences in the distributions of aerobes, facultative anaerobes, or obligate anaerobes compared to control mice (**Figure 5**).

## Discussion

There is emerging evidence that the ionic milieu in the intestine plays a critical role in inducing gut inflammation and colitis (Prasad and Visweswariah, 2021). Impaired Na^+^ and water transport, which occurs as a result of NHE3 deficiency, may mediate pathogenesis in gastrointestinal disorders such as CSD and IBD. It is becoming increasingly clear that the ionic milieu, alongside changes in luminal pH, can directly affect the composition of the gut microbiome. Several studies have shown that NHE3 deficiency predispose patients and animals to microbial dysbiosis and IBD (Engevik et al., 2013; Larmonier et al., 2013; Janecke et al., 2016; Laubitz et al., 2016; Harrison et al., 2018). We previously developed a novel inducible intestine-specific NHE3 knockout mouse that mimics symptoms of CSD, including alkaline diarrhea, increased luminal Na^+^ concentration, metabolic acidosis, hyponatremia and hyperkalemia (Xue et al., 2020). The aim of the current study was to determine how deletion of intestinal NHE3 in adulthood impacts the gut microbiome. We utilized metagenomic shotgun sequencing to study how the gut microbiome is altered from phyla to species levels with high resolution following inducible intestine-specific NHE3 deletion. Our data demonstrate that functional NHE3 is required for a healthy microbiome, and lack of intestinal NHE3 results in microbial changes that favor inflammation and downstream pathological consequences.

Changes to the intestinal microenvironment can lead to selective pressure on microbiota, ultimately leading to certain microbes gaining a growth advantage while others become restricted. This dysbiosis can make the host more susceptible to inflammatory or infectious diseases processes, which can further reshape the gut microbiome. Thus, it has become an area of interest to better understand how normal physiological functions, such as Na^+^/H^+^ exchange, can alter the microenvironment and therefore change the composition of the gut microbiome. Dysbiosis is typically characterized by a reduced overall microbial diversity (so called α-diversity) (Pickard et al., 2017). Indeed, patients with IBD have been shown to have less complex profiles of microbiota compared to healthy individuals (Ott et al., 2004). Similarly, conventional NHE3 deletion also resulted in reduced microbial diversity (Larmonier et al., 2013; Laubitz et al., 2016). Surprisingly, our study showed that inducible intestine-specific deletion of NHE3 results in higher microbial α-diversity compared with control mice. We speculate that increased diversity doesn’t necessarily reflect a healthy microbiome, but rather that the microenvironment has changed in such a way that there is expansion of less dominant pathogenic bacterial species. Importantly, differences in microbial diversity could also relate to animals being housed at difference facilities and/or fed different diets (Parker et al., 2018). In addition, we used an inducible NHE3^IEC-KO^ mouse model, in which NHE3 was deleted in the intestine during adulthood rather than NHE3 being absent throughout the entire lifespan. Given that colonization of microbiota begins at birth and can be derived from direct maternal transmission (including breast feeding), conventional NHE3 deletion could have a different effect on the microbiome compared with inducible NHE3 deletion in adult animals. Moreover, metagenomic shotgun sequencing provides high resolution at the species level which, when compared with previous studies using 16S rRNA sequencing, could specifically lead to differences in microbial α-diversity. Of note, we found that microbial β-diversity was significantly different in NHE3^IEC-KO^ compared with control mice, underscoring that the microbiome is distinctly different when NHE3 is deficient compared to healthy control mice.

The gut microbiome is predominantly composed of two bacterial phyla, *Bacteroidetes* and *Firmicutes*. NHE3^-/-^ mice have been shown to have higher *Bacteroidetes* abundance and reduced *Firmicutes* abundance in both the luminal and mucosa-associated microbiomes compared to wild-type littermates (Engevik et al., 2013; Larmonier et al., 2013; Harrison et al., 2018). Despite not seeing significant differences at the phylum level for the abundance of *Bacteroidetes* and *Firmicutes* in our study with inducible intestinal epithelial cell-specific NHE3 deletion, we did find that the *Firmicutes : Bacteroidetes* (F:B) ratio tended to be lower in NHE3^IEC-KO^ compared with control mice, which is consistent with previous studies demonstrating that a reduced F:B ratio favors IBD progression (Stojanov et al., 2020).

Even though we did not see differences in *Bacteroidetes* between genotypes, we did see significant expansion at the family (e.g., *Bacteroidaceae, Tannerellaceae, Rikenellaceae*), genus (e.g., *Bacteroides, Parabacteroides, Alistipes*), and species (e.g., *B. thetaiotamicron*, *P. distasonis*) levels in NHE3^IEC-KO^ compared with control mice. Given that NHE3^-/-^ (Schultheis et al., 1998) and NHE3^IEC-KO^ (Xue et al., 2020) mice have alkaline diarrhea, these findings are consistent with other studies that indicate *Bacteroidetes* thrive in conditions with a slightly higher pH (Ilhan et al., 2017). Of note, the *Bacteroides* member *B. thetaiotamicron* was also found to be increased in NHE3^-/-^ mice and was also found to be Na^+^ sensitive (Engevik et al., 2013). This suggests that the increased luminal salinity that occurs with NHE3 deficiency may give this microbe a competitive edge. Indeed, *B. thetaiotaomicron* has been identified as a pathobiont in models of IBD (Bloom et al., 2011; Hansen et al., 2012). The *Tannerellaceae* family has a relatively new genus *Parabacteroides*, which now has approximately 10 valid species, including *P. distasonis* (Ezeji et al., 2021). Recent studies have shown that *P. distasonis* has ambivalent effects in models of IBD, with reports of both pro-inflammatory and anti-inflammatory effects (Kverka et al., 2011). We found that *P. distasonis* was significantly higher in abundance in NHE3^IEC-KO^ compared with control mice; however, it remains unclear if this is beneficial or harmful. The genus *Alistipes* has recently emerged as having major implications in diseases such as IBD, cardiovascular disease, and cancer (Parker et al., 2020). In our study, we found that NHE3^IEC-KO^ mice had enrichments in 5 *Alistipes* species: *A. finegoldii*, *A. shahii*, *A. putredinis*, *A. obesi* and *A. senegalensis.* Interestingly, it has been shown that *A. finegoldii* attenuates colitis in mice (Dziarski et al., 2016).

Although *Firmicutes* have previously been shown to be contracted in NHE3^-/-^ mice and patients with IBD (Engevik et al., 2013; Larmonier et al., 2013), we did not see significant changes between genotypes at the phylum level. However, we did see a trend for contraction at the class (*Clostridia)* and order (*Clostridiales)* levels, and significant contraction at the family (*Lachnospiraceae*), genus (*Roseburia)*, and species (e.g., *C. scindens, R. hominis)* levels in NHE3^IEC-KO^ compared with control mice. The *Lachnospiraceae* and *Ruminococcaceae* families are known to be fairly abundant members of the normal gut microbiome; however, have been shown to be decreased in NHE3^-/-^ mice (Laubitz et al., 2016; Harrison et al., 2018). We found that *Lachnospiraceae* was contracted in NHE3^IEC-KO^ compared with control mice, but we did not find any differences in *Ruminococcaceae.* Reduced abundance of *Lachnospiraceae* correlates with decreased microbial production of short chain fatty acids (particularly butyrate), which serve as the primary fuel source for colonocytes and also exert immunomodulatory roles in the gut mucosa. Of note, *Lachnospiraceae* and *Ruminococcaceae* have been shown to be pH sensitive; a shift in pH from 5.5 to 6.5 dramatically stunted both microbial abundance and butyrate production (Walker et al., 2005). One of the most notable family members, *Roseburia*, resides in the mucus layer of the intestine (Van den Abbeele et al., 2013), making this microbe particularly vulnerable to changes in luminal pH as a result of dysfunctional Na^+^/H^+^ exchange. Our study and others have shown that NHE3 deficiency results in a dramatic reduction in the abundance of *Roseburia* (Harrison et al., 2018), which has also been seen in patients with IBD (Sellon et al., 1998; Chassaing et al., 2012). We observed more than a 3-fold reduction in *Roseburia* in NHE3^IEC-KO^ compared with control mice, which was predominantly attributed to the reduction of the species *R. hominis* (which accounts for >95% abundance of *Roseburia)*. Of note, a significant reduction in R. *hominis* in the intestine of ulcerative colitis patients was recently described (Patterson et al., 2017). Our data also revealed that other members within the family *Lachnospiraceae* are contracted in NHE3^IEC-KO^ mice, e.g., at the genus level, *Robinsoniella* was nearly depleted, 3-fold reduction in *Kineothrix*, nearly 2-fold reduction in *Lachnoclostridium* and *Butyrivibrio* (although did not reach statistical significance); and at the species level, e.g. *C. scindens* (protective against *Clostridium difficile* infection (Mills et al., 2018) and gut inflammation (Burgess et al., 2020))*, L. phytofermentans, K. alysoides, R. peoriensis, E. massiliensis*, and *C. polysaccharolyticum*.

The subdominant phylum *Proteobacteria* includes a wide variety of pathogens, and its expansion is a common risk factor for many diseases including IBD (Berry and Reinisch, 2013; Rizzatti et al., 2017). Our study and those in NHE3^-/-^ mice found a significant expansion in *Proteobacteria* (Engevik et al., 2013; Larmonier et al., 2013), which is consistent with our previous report that ~50% of NHE3^IEC-KO^ mice develop mild to moderate colitis three weeks after induction of intestinal NHE3 deletion (Xue et al., 2020). *Helicobacter* species have been shown to be endemic in mouse colonies and colonization with *H. ganmani* has been associated with increased release of proinflammatory cytokines in IL-10 deficient mice (Alvarado et al., 2015). We found that this species was absent in control mice, but present in NHE3^IEC-KO^ mice, suggesting it may play a role in the pathogenesis of intestinal disease.

Our study has several limitations, which warrant further studies. First, the microbiome composition can be drastically influenced by animal facility (Parker et al., 2018), so we cannot exclude facility-specific effects in our mouse colony. Replicating these microbiome experiments in animal facilities at other institutions would allow for confirmation that changes in the microbiome were specific to intestinal NHE3 deletion. Second, previous studies using microbial β-diversity analysis showed distinct signatures between mucosa-associated and fecal microbiota (Lo Presti et al., 2019). However, our study only evaluated changes in fecal microbiota. Future studies will further delineate the effect of loss of intestinal NHE3 on mucosa-associated microbiota. Third, only male mice were used in this study; whether or not there are sex differences in NHE3 deficiency-reshaped gut microbiota needs to be determined. Lastly, in mice, NHE3 is differentially expressed in distinct segments of small intestine (jejunum > duodenum > ileum) and colon (Fenton et al., 2017; Wang et al., 2019). Given that distinct segments of intestine harbor different microbial compositions, it remains to be determined if NHE3 modulates microbiota in a segment-specific way.

Taken together, our data provide high-resolution microbial composition analysis to dissect the differential abundance of microbes between NHE3^IEC-KO^ and control mice, elucidating the effects of intestinal NHE3 on gut microbiota. Overall, intestine-specific NHE3 deletion in adulthood creates an intestinal microenvironment in which certain inflammophilic species gain a competitive advantage over other anti-inflammatory species. Interestingly, microbiome analysis in patients with IBD demonstrated a reduction of obligate anaerobes and a sharp expansion of facultative anaerobes (Berry and Reinisch, 2013; Henson and Phalak, 2017). However, our data showed no change in aerobic/anaerobic microbial composition between NHE3^IEC-KO^ and control mice. The reason for this is not fully understood and warrants further analysis. Even though the causative relationship between NHE3 deficiency, dysbiosis, and IBD has not been completely understood to date, we have provided additional evidence that changes in the ionic milieu (Na^+^ and pH) as a result of intestine-specific deletion of NHE3 provides a competitive advantage for certain microbiota (e.g., *Bacteriodetes* and *Proteobacteria)* and a disadvantage for others (e.g., *Firmicutes).* This may predispose and/or promote pathogenesis of intestinal diseases such as IBD. In turn, the pathological state of IBD can further reshape the gut microbiota by influencing expansion of certain pathobionts that consequently inhibit NHE3 activity (Hayashi et al., 2004; Subramanya et al., 2007; Hodges et al., 2008), which reciprocally aggravates dysbiosis and IBD.

## Data Availability Statement

The data presented in the study are deposited in the Figshare repository (https://figshare.com/articles/dataset/Effect_of_intestinal_NHE3_deletion_on_microbiome/19358456).

## Ethics Statement

The animal study was reviewed and approved by University of South Florida Institutional Animal Care and Use Committee.

## Author Contributions

TR, JDR and JX conceived and designed the work. TR, JDR, JX, LT and JW contributed to the acquisition, analysis, or interpretation of data for the work. TR, JDR and JX drafted the work; TR, JDR, JX, LT and JW revised it critically for important intellectual content. TR, JDR, JX, LT and JW approved the final version of the manuscript. All authors contributed to the article and approved the submitted version.

## Funding

This work was supported by the National Institute of Diabetes and Digestive and Kidney Diseases grant 1R01DK110621 (to Dr. Rieg), VA Merit Review Award IBX004968A (to Dr. Rieg) and an American Heart Association Transformational Research Award 19TPA34850116 (to Dr. Rieg). Financial support for this work was also provided by the NIDDK Diabetic Complications Consortium (RRID : SCR_001415, www.diacomp.org), grants DK076169 and DK115255 (to Dr. Rieg). Dr. Thomas by an American Heart Association postdoctoral fellowship (828731).

## Conflict of Interest

JW discloses equity ownership in Resphera Biosciences LLC.

The remaining authors declare that the research was conducted in the absence of any commercial or financial relationships that could be construed as a potential conflict of interest.

## Publisher’s Note

All claims expressed in this article are solely those of the authors and do not necessarily represent those of their affiliated organizations, or those of the publisher, the editors and the reviewers. Any product that may be evaluated in this article, or claim that may be made by its manufacturer, is not guaranteed or endorsed by the publisher.
